# New classification of *HLA-DRB1 *alleles in rheumatoid arthritis susceptibility: a combined analysis of worldwide samples

**DOI:** 10.1186/ar2379

**Published:** 2008-02-28

**Authors:** Thomas Barnetche, Arnaud Constantin, Alain Cantagrel, Anne Cambon-Thomsen, Pierre-Antoine Gourraud

**Affiliations:** 1 Department of Epidemiology and Public Health, UMR Inserm U 558, University Paul Sabatier Toulouse III, Faculty of Medicine Purpan, 37 Allées Jules Guesde, Toulouse cedex 7, 31073, France; 2 Rheumatology Department, Larrey University Hospital, 24 chemin de Pouvourville, Toulouse cedex 9, 31059, France; 3 JE2510, University Paul Sabatier Toulouse III, 118 route de Narbonne, Toulouse, 31062 cedex 9, France; 4 UF de Méthodologie de la recherche clinique, Epidemioloy Unit, Toulouse University Hospital, 37 Allées Jules Guesde, Toulouse cedex 7, 31073, France

## Abstract

**Introduction:**

Rheumatoid arthritis (RA) is a complex polygenic disease of unknown etiology. *HLA-DRB1 *alleles encoding the shared epitope (SE) (RAA amino acid pattern in positions 72 to 74 of the third hypervariable region of the DRβ1 chain) are associated with RA susceptibility. A new classification of *HLA-DRB1 *SE alleles has been developed by Tezenas du Montcel and colleagues to refine the association between *HLA-DRB1 *and RA. In the present study, we used RA samples collected worldwide to investigate the relevance of this new *HLA-DRB1 *classification in terms of RA susceptibility across various Caucasoid and non-Caucasoid patients.

**Methods:**

Eighteen subsamples were defined from a total number of 759 cases and 789 controls and grouped in 10 samples on the basis of their ethnic origin. *HLA-DRB1 *alleles were divided into five groups (S_1_, S_2_, S_3D_, S_3P_, and X) according to the new *HLA-DRB1 *allele classification. The whole analysis was performed by comparing carrier frequencies for the five HLA-DRB1 allele groups between RA patients and controls across the 10 Caucasoid and non-Caucasoid samples. The Mantel-Haenszel method of meta-analysis provided a global odds ratio (OR) estimate with 95% confidence interval (CI).

**Results:**

A positive association with RA susceptibility was found for S_2 _allele carriers (OR 2.15, 95% CI 1.54 to 3.00; *p *< 10^-5^) and S_3P _allele carriers (OR 2.74, 95% CI 2.01 to 3.74; *p *< 10^-5^). A negative association was found for S_1 _alleles (OR 0.60, 95% CI 0.48 to 0.76; *p *< 10^-4^) and X alleles (OR 0.58, 95% CI 0.39 to 0.84; *p *= 4 × 10^-3^). No significant association was highlighted for the S_3D _group of alleles (OR 0.89, 95% CI 0.69 to 1.14; *p *= 0.89). The complementary genotype analysis fit with the genotype risk hierarchy previously reported in Caucasoid RA patients.

**Conclusion:**

So far, the present study is the first attempt to investigate the relevance of this new *HLA-DRB1 *classification in terms of RA susceptibility on both Caucasoid and non-Caucasoid samples. Our results support the hypothesis of a differential role played by different *HLA-DRB1 *allele groups in RA susceptibility across different ethnic backgrounds and confirm the interest of such an *HLA-DRB1 *classification in differentiating predisposing and protective alleles.

## Introduction

Rheumatoid arthritis (RA) is the most frequent chronic inflammatory rheumatic disease in the world, with prevalence estimates of 0.25% to 0.5%. Its pathogenesis is multifactorial and genetic factors may contribute for 40% to 60% of the total risk [1]. Among possible genetic factors, the *HLA-DRB1 *gene appears clearly associated with RA [2]. This association was first suggested more than 30 years ago [3] and was elaborated 10 years later by Gregersen and colleagues [4], who demonstrated that RA was associated with several *HLA-DRB1 *alleles (DRB1*0101, DRB1*0102, DRB1*0401, DRB1*0404, DRB1*0405, DRB1*0408, DRB1*1001, and DRB1*1402) encoding the RAA sequence of amino acids at positions 72 to 74 in the third hypervariable region of the *DRβ1 *chain, known as the shared epitope (SE). Despite significant improvement in molecular biology techniques, association mechanisms between HLA-DRB1*SE^+ ^alleles and RA remain debated and authors have demonstrated that each SE allele does not confer the same risk [4-6].

In a more recent study, Tezenas du Montcel and colleagues [8] advanced a new classification of *HLA-DRB1 *alleles, reconsidering the SE model in RA susceptibility. According to this new classification, the susceptibility to RA, which depends on whether the RAA sequence occupies positions 72 to 74, was modulated by the amino acids at positions 70 and 71, which led to the definition of five groups of *HLA-DRB1 *alleles: S_1_, S_2_, S_3P_, S_3D_, and X alleles. Michou and colleagues [9] tested and validated this new classification in an independent sample of 100 French Caucasoid RA trio families, providing estimates for the susceptibility risk genotypes. In the present study, we used worldwide RA samples from the 13th International Histocompatibility Working Group (IHWG) to investigate the relevance of this new *HLA-DRB1 *allele classification in terms of RA susceptibility across various Caucasoid and non-Caucasoid population samples.

## Materials and methods

### Selection process of case and control population samples

RA cases and healthy controls included in the present study were selected from a population of 2,376 individuals (1,210 cases and 1,166 controls), initially gathered by 19 laboratories in 17 countries in the framework of the 13th IHWG. The data are publicly available from the dbMHC (Major Histocompatibility Complex database) website of the National Center for Biotechnology Information (Bethesda, MD, USA) [7]. All RA cases met the following criteria: adult onset RA (by definition, 16 years of age or older) and the American College of Rheumatology criteria for RA [8]. For each laboratory, healthy controls were selected within the same geographical area as the RA cases. A selection procedure of cases and controls was carried out in order to allow the comparison of the data issued from the different laboratories that participated in the 13th IHWG: (a) cases and controls of undocumented origin were excluded, (b) samples consisting of cases without controls and samples of less than 20 individuals were discarded, and (c) cases and controls that were matched beforehand for specific *HLA-DRB1*–*HLA-DQB1 *haplotypes were excluded. Data from different submitters consisting of individuals from the same origin were pooled when no significant departures were found as assessed by an admixture test, which asymptotically follows a chi-square distribution with 1 degree of freedom. According to this selection procedure, 758 cases and 789 controls, issued from 10 different ethnic origin subsamples, were included in the present study (Table 1).

**Table 1:** Composition of the selected rheumatoid arthritis case and control population samples

Ancestry	Sample origin	Number of cases	Number of controls
Caucasian	Greek	37	31
Caucasian	Spanish	141	234
Caucasian	American (Whites)	206	105
Amerindian	North American (Amerinds)	98	78
African-American	North American (Blacks)	46	51
African	San (Bushman)	23	57
Asian	Korean	81	68
Asian	Chinese	21	20
Asian	Javanese	66	72
Slavic	Russian	40	73
Total		759	789

These data were extracted from the 13th International Histocompatibility Working Group rheumatoid arthritis samples and are available on the National Center for Biotechnology Information website [28]. For the sample selection procedure, please refer to the Materials and methods section of this article.

### *HLA-DRB1 *genotyping

All RA cases and controls were genotyped for *HLA-DRB1*. *HLA-DRB1 *typing techniques used in the framework of the 13th IHWG are described extensively in the 13th IHWG Proceedings [9,10].

### *HLA-DRB1 *classification

*HLA-DRB1 *alleles were divided into five groups according to the classification proposed by Tezenas du Montcel and colleagues [11,12]. Briefly, the *HLA-DRB1 *alleles were first divided into two groups according to the presence or absence of the RAA sequence at positions 72 to 74 and were denoted S and X alleles, respectively. The S alleles were subsequently divided into three groups according to the amino acid (alanine [A], glutamic acid [E], lysine [K], or arginine [R]) at position 71: S_1 _for ARAA and ERAA, S_2 _for KRAA, and S_3 _for RRAA. Since an aspartic acid (D) at position 70 was reported to be protective against RA in contrast to a glutamine (Q) or an arginine (R) at the same position [13], two additional groups were defined: S_3D _for DRRAA and S_3P _for QRRAA or RRRAA [11,12].

### Statistical analysis

To identify association with RA susceptibility, odds ratios (ORs) were calculated for the presence of the S_1_, S_2_, S_3P_, S_3D_, and X alleles. Confidence intervals (CIs) are given at 95% confidence. Consistently with previous findings [7-9] and with the main objective of this work (which is to challenge these previous findings in various Caucasoid and non-Caucasoid populations), we performed the whole analysis under a dominant effect model by comparing carrier frequencies for the different *HLA-DRB1 *allele groups defined according to the classification between RA patients and controls across the 10 Caucasoid and non-Caucasoid samples.

We used a meta-analysis approach to combine the data issued from the different laboratories that participated in the 13th IHWG. The Mantel-Haenszel method assumes a fixed effect and combines studies using a method similar to inverse variance approaches to determine the weight given to each study. It provides a common OR estimate, taking into account the weight of the different samples and 95% CI. OR and 95% CI are shown on forest plots for each allele group studied. Statistical heterogeneity of the considered samples was assessed on the basis of the Q test (chi-square), using a significance level of 0.05, and reported with the I^2 ^statistic (in which high values indicate high heterogeneity). An I^2 ^value of greater than 50% was considered the threshold for heterogeneity. Genotype risk analyses were conducted using the same method. All computations were performed using the Revman 4.2.8 software package developed by the Nordic Cochrane Center (Copenhagen, Denmark) [14] and Stata version 7.0 software (StataCorp LP, College Station, TX, USA). All *p *values were two-sided. *P *values of less than 0.05 were considered significant, and corrections for multiple testing were mentioned when relevant.

## Results

### Carrier frequencies of the different *HLA-DRB1 *allele groups in RA cases and controls for the various Caucasoid and non-Caucasoid population samples

**Figure 1:**
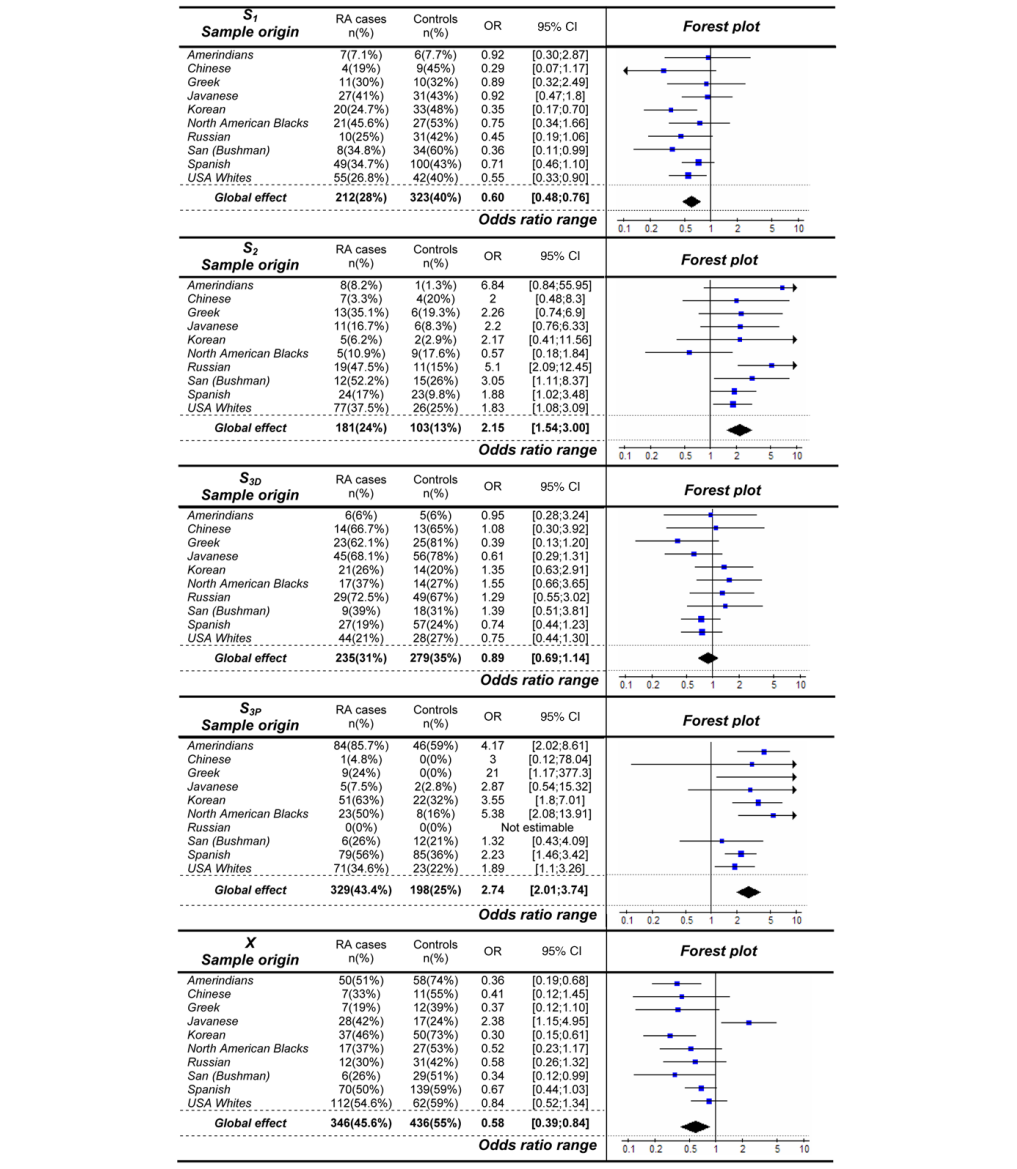
Carrier frequency comparisons of the different HLA-DRB1 allele groups between rheumatoid arthritis (RA) cases and controls across the various Caucasoid and non-Caucasoid population samples and overall effect estimation. This figure provides a summary meta-analysis of allele carrier frequencies according to HLA-DRB1 allele classification, in selected samples among the data available from the 13th International Histocompatibility Working Group on Rheumatoid Arthritis. For each population sample, odds ratios (ORs) and 95% confidence intervals (95% CIs) evaluate the significance of the association between the different HLA-DRB1 allele groups and RA susceptibility (blue boxes). The combined ORs and 95% CIs evaluate the significance of the global effect of the different HLA-DRB1 allele groups on RA susceptibility over all population samples. *P *values were calculated with the Mantel-Haenszel method (black diamonds).

Figure 1 shows the carrier frequencies for the different *HLA-DRB1 *allele groups, as defined according to the classification developed by Tezenas du Montcel and colleagues [11], in cases and controls of each sample selected from the 13th IHWG. No significant departures from Hardy-Weinberg equilibrium were observed (all *p *> 0.05 after correction for multiple testing). Statistical testing for heterogeneity in the X allele group revealed a significant difference between samples (I^2 ^= 62.9%, *p *= 4 × 10^-3^). No significant heterogeneity could be observed for the S_1 _(I^2 ^= 0%, *p *= 0.57), S_2 _(I^2 ^= 15.9%, *p *= 0.30), S_3P _(I^2 ^= 19.5%, *p *= 0.27), or S_3D _(I^2 ^= 23.6%, *p *= 0.23) groups of *HLA-DRB1 *alleles.

### Carrier frequency comparisons of the different *HLA-DRB1 *allele groups between RA cases and controls across the various Caucasoid and non-Caucasoid population samples and overall effect estimation

Results of allele carrier frequency comparisons between RA cases and controls across the various Caucasoid and non-Caucasoid population samples are presented in Figure 1. An overall positive association with RA susceptibility was found for S_2 _alleles (OR 2.15, 95% CI 1.54 to 3.00; *p *< 10^-5^) and S_3P _alleles (OR 2.74, 95% CI 2.01 to 3.74; *p *< 10^-5^). An overall negative association with RA susceptibility was highlighted for S_1 _alleles (OR 0.60, 95% CI 0.48 to 0.76; *p *< 10^-4^) and X alleles (OR 0.58, 95% CI 0.39 to 0.84; *p *= 4 × 10^-3^). No significant association with RA susceptibility was found for the S_3D _group of alleles (OR 0.89, 95% CI 0.69 to 1.14; *p *= 0.88). In such an analysis, a potential bias may be introduced by the presence of allele adverse effect in the control group. For example, in the analysis of the S_2 _effect, the association may be overestimated due to the presence of S_3D _carriers in the control group (noncarrier of S_2_). Similarly, the effect of S_2 _may be underestimated thanks to the presence of S_3P _carriers. After controlling for the adverse effect of S_3D _and S_1 _in the analysis of S_2_, the association with RA susceptibility remained significant (*p *< 0.05).

### Carrier frequency comparisons of the different *HLA-DRB1 *allele groups between RA cases and controls in Caucasoid and non-Caucasoid population samples

Results of allele carrier frequency comparisons between RA cases and controls in Caucasoid and non-Caucasoid population samples are presented in Table 2. In the Caucasoid population sample, S_2 _alleles (OR 2.61, 95% CI 1.87 to 3.64) and S_3P _alleles (OR 1.86, 95% CI 1.39 to 2.49) were positively associated with RA susceptibility, whereas S_1 _alleles (OR 0.59, 95% CI 0.45 to 0.79) and X alleles (OR 0.74, 95% CI 0.56 to 0.96) were negatively associated with RA susceptibility. In the non-Caucasoid population sample, S_3P _alleles (OR 2.93, 95% CI 2.21 to 4.04) were positively associated with RA susceptibility, whereas S_1 _alleles (OR 0.52, 95% CI 0.37 to 0.71) and X alleles (OR 0.61, 95% CI 0.45 to 0.83) were negatively associated with RA susceptibility.

**Table 2:** Carrier frequency comparisons of the different HLA-DRB1 allele groups between rheumatoid arthritis cases and controls in Caucasoid and non-Caucasoid population samples

	RA cases, n (%)	Controls, n (%)	OR (95% CI)
S_1 _alleles			
Caucasoids	125 (29.4%)	183 (41.3%)	0.59 (0.45–0.79)
Non-Caucasoids	87 (26%)	140 (40%)	0.52 (0.37–0.71)
S_2 _alleles			
Caucasoids	133 (31%)	66 (15%)	2.61 (1.87–3.64)
Non-Caucasoids	48 (14%)	37 (11%)	1.40 (0.88–2.21)
S_3D _alleles			
Caucasoids	123 (29%)	159 (36%)	0.73 (0.55–0.97)
Non-Caucasoids	112 (33%)	120 (35%)	0.95 (0.69–1.30)
S_3P _alleles			
Caucasoids	159 (37.5%)	108 (24%)	1.86 (1.39–2.49)
Non-Caucasoids	170 (51%)	90 (26%)	2.93 (2.12–4.04)
X alleles			
Caucasoids	201 (47%)	244 (55%)	0.74 (0.56–0.96)
Non-Caucasoids	145 (43%)	192 (55%)	0.61 (0.45–0.83)

The Caucasoid sample population refers to the combination of the following population samples: Greek, Spanish, Russian, and American (Whites). The non-Caucasoid sample population refers to the combination of the following population samples: North American (Amerinds), North American (Blacks), Bushmen, Korean, Chinese, and Javanese. The combined odds ratios (ORs) and 95% confidence intervals (CIs) evaluate the significance of the global effect of the different HLA-DRB1 allele groups on rheumatoid arthritis (RA) susceptibility in Caucasoids and non-Caucasoids.

### Overall effect estimation of genotypes resulting from the classification of HLA-DRB1 alleles on RA susceptibility

Using the approach proposed by Michou and colleagues [9], we further pooled the three low-risk allele groups (S_1_, S_3D_, and X), thus producing a new grouping called L alleles. Thus, in subsequent analyses, we considered only three allele groups (S_2_, S_3P_, and L alleles), with six corresponding genotypes [12]. The results of observed genotype distributions and of genotype relative risks are shown in Table 3. S_2_/S_3P _and S_3P_/S_3P _were associated with the greatest risks for RA, with ORs (95% CIs) of 7.25 (3.26 to 16.14) and 5.15 (2.91 to 9.12), respectively. These are followed by S_2_/S_2_, S_2_/L, and S_3P_/L, with ORs (95% CIs) of 4.95 (2.2 to 11.18), 2.41 (1.60 to 3.65), and 2.33 (1.57 to 3.45), respectively. These analyses were all performed using the L/L genotype as reference.

**Table 3:** Overall effect estimation of genotypes resulting from the classification of HLA-DRB1 alleles on rheumatoid arthritis susceptibility

Genotypes	Genotype distribution, n (%)		OR (95% CI)	*P *values
			
	RA cases, *n *= 758	Controls, *n *= 789		
S_2_/S_3P_	39 (5.1%)	7 (0.9%)	7.25 (3.26–16.14)	<10^-5^
S_3P_/S_3P_	74 (9.8%)	31 (4%)	5.15 (2.91–9.12)	<10^-5^
S_2_/S_2_	24 (3.2%)	8 (1%)	4.95 (2.2–11.18)	<10^-4^
S_2_/L	121 (16%)	78 (9.9%)	2.41 (1.60–3.65)	<10^-4^
S_3P_/L	179 (23.6%)	136 (17.2%)	2.33 (1.57–3.45)	<10^-4^
L/L	321 (42.3%)	529 (67%)	1	

S_1_, S_2_, S_3P_, S_3D_, and X allele groups were defined according to the amino acid sequence at positions 70 to 74. According to the approach proposed by Michou and colleagues [9], we pooled the three low-risk allele groups (S_1_, S_3D_, and X), so called L alleles. Thus, in subsequent analyses, we considered only three allele groups (S_2_, S_3P_, and L alleles), with six corresponding genotypes [12]. The reference genotype is L/L. The combined odds ratios (ORs) and 95% confidence intervals (CIs) evaluate the significance of the global effect of the different HLA-DRB1 genotype groups on rheumatoid arthritis (RA) susceptibility over all population samples. *P *values were calculated with the Mantel-Haenszel method.

## Discussion

In the present association study, we investigated the relevance of the classification of *HLA-DRB1 *alleles proposed by Tezenas du Montcel and colleagues [11] regarding susceptibility to RA, across various Caucasoid and non-Caucasoid population samples, using publicly available data from the 13th IHWG RA studies. Across these various population samples, our approach strengthens the relevance of this classification, exhibiting an overall positive association with RA susceptibility for S_2 _and S_3P _alleles and an overall negative association with RA susceptibility for S_1 _and X alleles. The genotype analysis performed in the present study fits with the genotype risk hierarchy previously reported in Caucasoid RA sporadic cases [11] and families [12].

The present combined analysis included 10 samples from different genetic backgrounds. Although we did not observe significant heterogeneity for S_1_, S_2_, S_3D _and S_3P _allele groups, we observed significant heterogeneity for the X allele group across the different population samples. The fixed effect model of the Mantel-Haenszel method, used for the overall effect analysis of the *HLA-DRB1 *allele and genotype groups on RA susceptibility in the present study, assumes that each allele group carries out a homogeneous effect on RA susceptibility across the various Caucasoid and non-Caucasoid samples. The heterogeneity observed for the X allele group may be questioned according the heterogeneity of the *HLA-DRB1 *allele and genotype groups at two levels across the different population samples: the effect level and the frequency level. Our data suggest that there is a differential effect of the S_1_, S_2_, S_3D _and S_3P _allele groups on RA susceptibility. Each of these effects seems homogenous across the various population samples. Because the SE allele distribution varies across these populations, the resulting effect of the X allele group on RA susceptibility depends both on the frequency of the S_1_, S_2_, S_3D _and S_3P _allele groups, and their respective effects on RA susceptibility, which might explain the observed heterogeneity of the effect of the X allele group in our study.

The contribution of SE alleles to RA susceptibility has been confirmed by numerous studies on different populations. For example, a recent meta-analysis on Latin American RA patients has shown the important role played by SE in RA susceptibility [15]. However, RA prevalence studies have shown differences in frequency estimations between populations with different genetic backgrounds. The highest prevalence rates have been found in Native American populations with estimation ranges of 32 to 48 per 1,000 men and 59 to 70 per 1,000 women. In Afro-Caribbean people who live in the UK, RA prevalence appeared to be lower than that in the general population. In urban African populations, RA prevalence was estimated around 10 per 1,000 and was found to be significantly higher than in rural populations. Studies on Chinese populations have reported lower prevalence estimations than in European ones. Molokhia and McKeigue previously pointed out the difficulty brought up by admixture in investigating the etiology of rheumatic diseases, notably for RA [16]. The significant variations observed in the incidence and prevalence of RA among different populations or ethnic groups could be explained, in part, by genetic variations in the HLA region, especially variations in the prevalence of SE in different populations [17,18]. In addition, as no consideration of environmental exposure variations between the population samples studied was made, the heterogeneity could be explained by the different impact of environmental factors on RA susceptibility in each different sample, such as nutrition as previously suggested, in particular in the Greek population [18,19]. In addition to nutrition, environmental factors such as exposure to cigarette smoking [20,21] or individual factors such as gender [22] may influence susceptibility to RA by interacting with genetic factors such as *HLA-DRB1*.

The classification proposed by Tezenas du Montcel and colleagues [11], based on amino acid sequence at positions 70 to 74, does not aim to account for all previously reported associations between particular HLA-DRB1 alleles and RA susceptibility in specific ethnic backgrounds. For example, the previously reported association between the HLA-DRB1*0901 allele and RA susceptibility in East Asian populations could not be tested in the present study, as this particular allele was classified together with many others as an X allele [23-25]. The high frequency of the HLA-DRB1*0901 allele in the Javanese population could contribute both to the association found between X alleles and susceptibility to RA in this particular population sample and to the observed heterogeneity of the X allele group.

The contribution of the *HLA-DRB1 *allele classification in accounting for the genetic contribution of the *HLA-DRB1 *gene was previously analyzed in terms of RA severity and in terms of autoantibody production such as anti-cyclic citrullinated peptide (anti-CCP) antibodies and anti-deiminated human fibrinogen autoantibodies. As RA severity outcomes as well as anti-CPP information were not collected in the framework of the 13th IHWG, we were not able to discuss the relevance of the classification of HLA-DRB1 alleles proposed by Tezenas du Montcel and colleagues [8] regarding RA severity or autoantibody production in the various Caucasoid and non-Caucasoid population samples included in the present study.

## Conclusion

Across these various samples coming from both Caucasoid and non-Caucasoid populations, we investigated the relevance of the classification of HLA-DRB1 alleles proposed by Tezenas du Montcel and colleagues [11] regarding susceptibility to RA. We confirm previous findings on the contribution of the S_2 _and S_3P _risk allele groups to RA susceptibility. In spite of the small sample size in some ethnic groups, the present study allows the differentiation between predisposing and protective HLA-DRB1 SE alleles in both Caucasoid and non-Caucasoid RA patients.

This report also emphasized the very crucial importance of public release of large-scale study data in genetic epidemiology. The need for large samples to refine the study of effects of modest magnitude and the necessity to replicate studies across different ethnic backgrounds rely on easy access to a large variety of data organized in a systematic way. After an initial period of restricted use of the data by the initial investigators, the access to clinical and genetic anonymous individual data should be made possible; this is the current policy of the National Institutes of Health (Bethesda, MD, USA) for genome-wide association study results [26]. Combined with a detailed description of the sampling scheme for both patients and controls, advanced statistical analysis will contribute to enhance secondary uses of data valorizing the efforts of previously completed studies [27].

## Abbreviations

Anti-CCP = anti-cyclic citrullinated peptide; CI = confidence interval; IHWG = International Histocompatibility Working Group; OR = odds ratio; RA = rheumatoid arthritis; SE = shared epitope.

## Competing interests

The authors declare that they have no competing interests.

## Authors' contributions

TB, ACo, and P-AG took the leadership of the study in both immunological and statistical aspects. ACa contributed through the assessment of clinical aspects. AC-T contributed to the statistical analysis. All authors read and approved the final manuscript.
